# Supplementary material for: Transition from predictable to variable motor cortex and striatal ensemble patterning during behavioral exploration
Source: Nat Commun. 2022 May 4;13:2450. doi: 10.1038/s41467-022-30069-1 (PMC9068924; doi:10.1038/s41467-022-30069-1)
Supplement: Supplementary file 1 — Supplementary Information [file 41467_2022_30069_MOESM1_ESM.pdf]

SUPPLEMENTARY INFORMATION

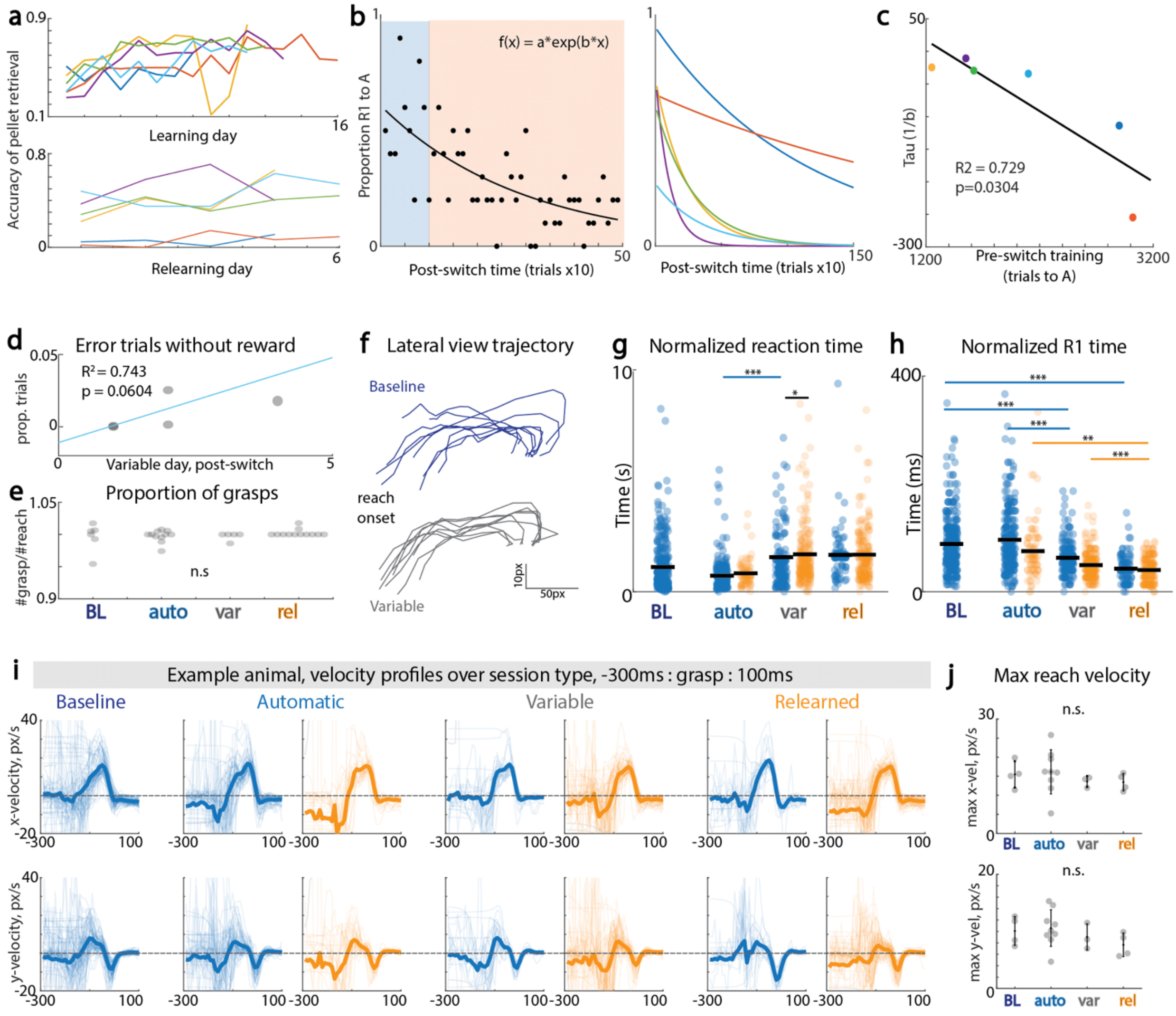

Supp. Fig. 1 | Relearning is a multi-day process with preservation of velocity.
a. Learning curves for reaching to position A, prior to switching location (top) and relearning curves for success in obtaining pellet at B (bottom). **b.** Proportion of reaches to A, per 10 trials, with exponential decay curve fit, for example animal (left), and across animals (right). **c.** Slope of decay, tau, versus trials of training to A prior to switch. **d.** Proportion of total learning trials to A where pellet was erroneously not present as compared to timing of variable session post-switch. **e.** Proportion of grasps relative to reaches for each session, by session type. **f.** Example first reach trajectories, from reach onset to endpoint, for baseline session (top) and variable session (bottom). **g.** Normalized† reaction time, as defined by time from trial start to reach start, for all trials across session types. **h.** Normalized† first reach to first grasp time across animals and session types. **i.** Velocity profiles 300ms before to 100ms after first grasp, in the x-direction (top) and y-direction (bottom) across A reaches (blue) and B reaches (orange), across session types for an example animal. **j.** Maximum reach velocity for trial-averaged velocity profile, across all sessions from all animals with lateral view video, in the x-direction (top) and y-direction (bottom). Max reach velocity: BL,

n=4 sessions; auto, n=9 sessions; var, n=3 sessions; rel, n=4 sessions. Data are presented as mean values +/- SD.

† normalization was performed by subtracting the minimum value across all trials of all sessions for an animal from the raw values.

* < 0.05, ** < 0.01, ***<0.001, linear mixed effects model, Bonferroni-corrected.

† normalization was performed by subtracting the minimum value across all trials of all sessions for an animal from the raw values.

* < 0.05, ** < 0.01, ***<0.001, linear mixed effects model, Bonferroni-corrected.

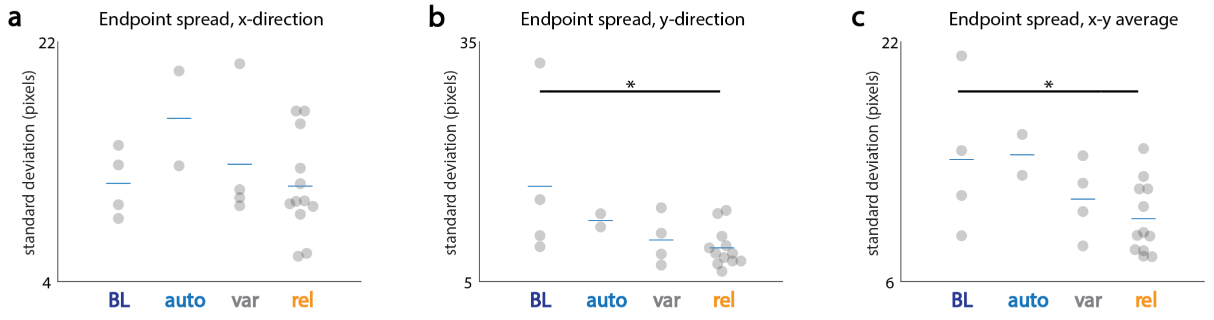

Supp. Fig. 2 | Variability in endpoints remains relatively preserved across session types. a. Standard deviation in the x-direction of reach endpoints, where each dot represents a single session from a single animal, all sessions included. **b.** Same as (a), for standard deviation in the y-direction. **c.** Average of x- and y- standard deviation of reach endpoints for a session, all sessions across animals included.
* < 0.05, linear mixed effects model, Bonferroni-corrected.

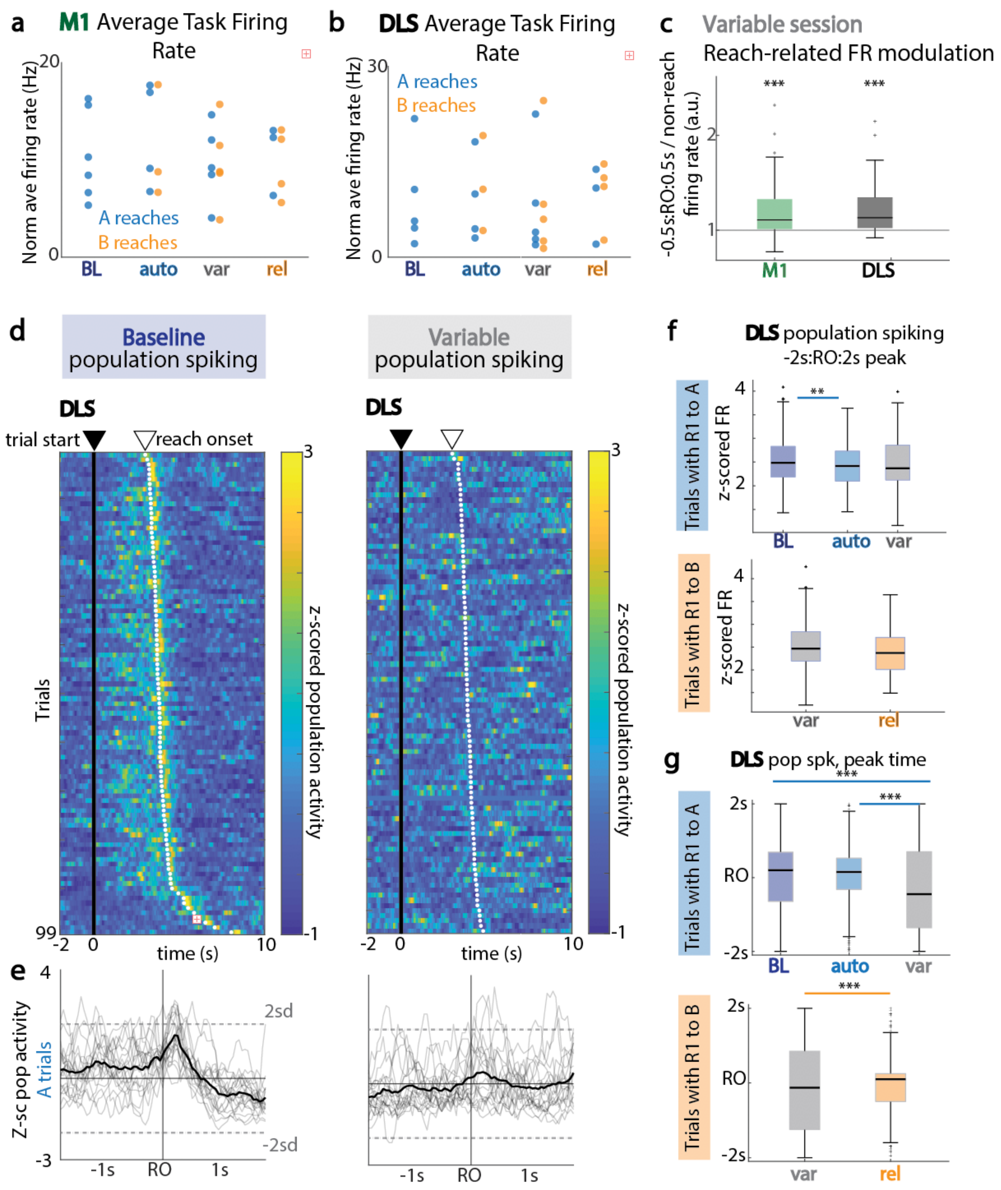

Supp. Fig. 3 | Higher order spiking structure is preserved in M1 and DLS, with temporal variability in spiking patterns.

a. Average firing rate for all M1 units, normalization via median of 1000-fold sub-sampling of neurons within a session, per animal for A reaches (blue) and B reaches (orange) for -2s to 2s around reach onset. **b.** Same as a, for DLS units per animals, across session types. **c.** Unit modulation during reach period, -0.5s to 0.5s around reach onset, relative to non-reach periods, for M1 (green) and DLS (black) units, two-sided paired t-test. M1 FR modulation, n=72 units; DLS FR modulation, n=58 units. Data are presented as box plots with 25th, 50th, and 75th percentiles. **d**. DLS z-scored population spiking activity across entire trial, for all trials in a baseline session (left) and variable session (right) for an example animal. **e.** Trial DLS population modulation, example A reach trials, z-scored population spiking for -2s to 2s around first reach onset for baseline session (left) and variable session (right). **f.** Time of peak population spiking within -2s to 2s around first reach onset in DLS units for trials with first reach to A (top) and trials with first reach to B (bottom). R1 to A: BL, n=291 trials; auto, n=256 trials; var, n=169 trials. R1 to B: var,

n=226 trials; rel, n=214 trials. Data are presented as box plots with 25th, 50th, and 75th percentiles. **g.** DLS single-trial population spiking modulation within -2s to 2s around first reach onset, for the same trials as in **f**, with first reach to A (top) and to B (bottom). Data are presented as box plots with 25th, 50th, and 75th percentiles.

* < 0.05, ** < 0.01, ***<0.001, c) two-sided paired t-test; f,g) Bartlett's test for homoscedasticity.

a

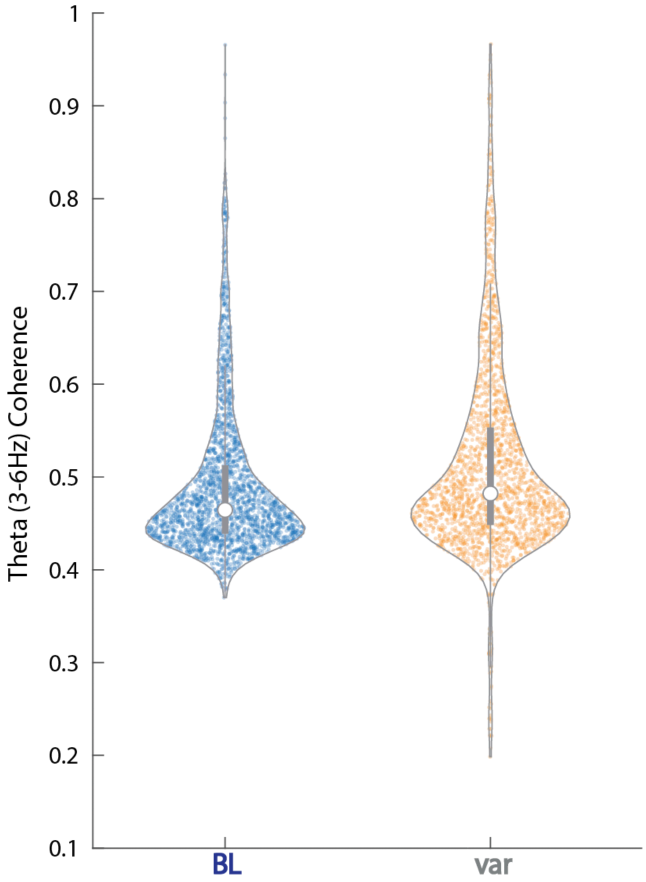

Supp. Fig. 4 | Stability of 3-6Hz M1-DLS coherence during variable state.

a. Single trial average 3-6Hz coherence values for -250ms:RO:250ms across animals, for trials with first reach to A during baseline sessions (left, blue) and trials with first reach to B for variable session (right, orange). Only one session per animal for each session type was included. BL, n=2360 M1-DLS unit pairs; var, n=1936 M1-DLS unit pairs. No significant difference in distributions, linear mixed effects model.

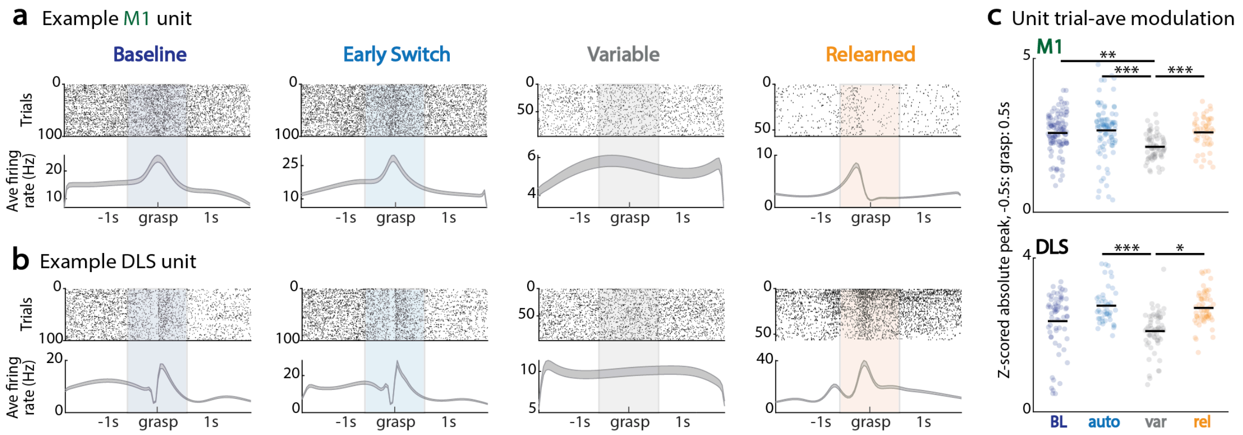

Supp. Fig. 5 | Loss of consistent grasp-locked M1 and DLS neural spiking during relearning

a. Top: Rasters for a single M1 unit† across sessions (same channel across days). Bottom: trial-averaged firing rate (mean ± SEM) for -2s to 2s around first reach onset. **b**. Same as (a), for a DLS unit.† **c.** (top) M1 unit peak trial-averaged modulation around first grasp, across session types; peak modulation was calculated after z-scoring unit firing across the session; (bottom) **s**ame as (top), for DLS units.

†Unit may not be the same across sessions.

* < 0.05, ** < 0.01, ***<0.001, linear mixed effects model, Bonferroni-corrected.

Source data are provided as a Source Data file.

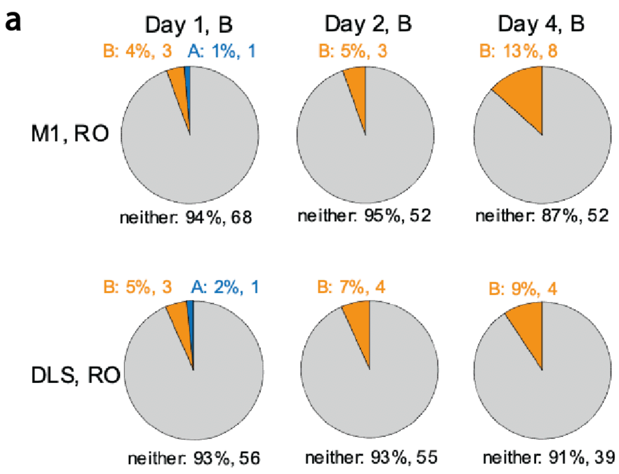

Supp. Fig. 6 | Majority of M1 and DLS spiking not related to reach direction.
a. Top: proportion of M1 units that are significantly tuned to A or B direction reach, for sessions after pellet location has been switched to B, grouping all units across animals. Bottom: same as above, for DLS units.

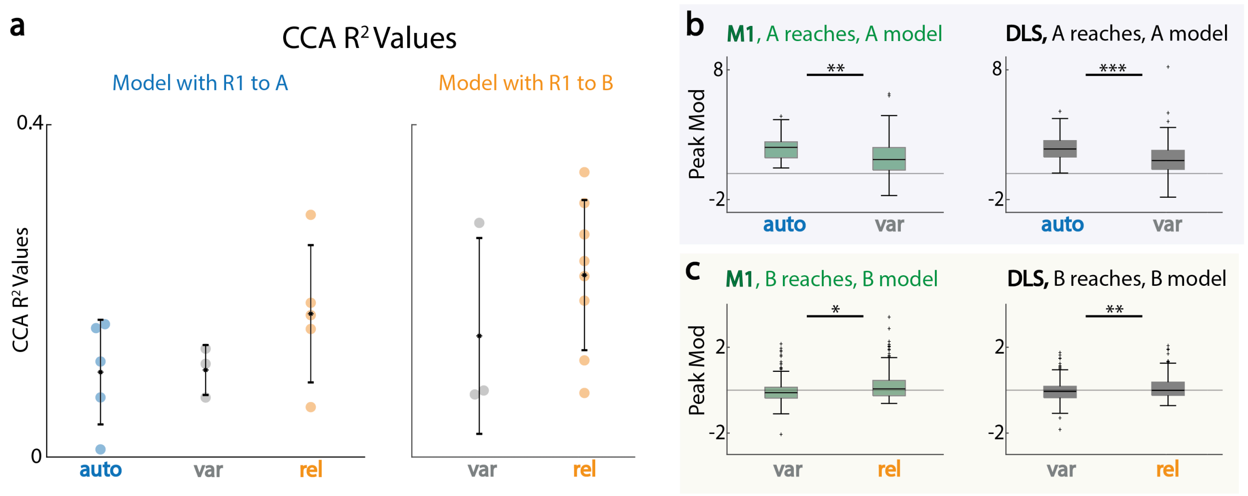

Supp. Fig. 7 | M1-DLS CCA subspace analysis.

a. Comparison of R^2 values for significant CCA models fit on trials with first reach to A across session types (left) and trials with first reach to B across session types (right). Each point represents the R^2 value for the top significant CV from a single session, across all sessions that met CCA inclusion criteria. R1 to A: auto, n=5 CCA models; var, n=3 CCA models; rel, n=5 CCA models. R1 to B: var, n=3 CCA models; rel, n=8 CCA models. Data are presented as mean values +/- SD. **b.** Reach-specific epoch CCA model, peak subspace activation between -200ms :RO:200ms for trials with first reach to A, for M1 activations (left, green) and DLS activations (right, black), across automatic and variable sessions, for CCA models built on the reach-related epoch of -0.5s to 0.5s around reach onset. R1 to A: auto, n=135 trials; var, n=95 trials. Data are presented as box plots with 25th, 50th, and 75th percentiles. **c.** Same as (b), for trials with first reach to B, across variable and relearned sessions. R1 to B: var, n=150 trials; rel, n=416 trials. Data are presented as box plots with 25th, 50th, and 75th percentiles.

*<0.05, **<0.01, ***<0.001, linear mixed effects model, Bonferroni-corrected.
