# Supplementary material for: Transition from predictable to variable motor cortex and striatal ensemble patterning during behavioral exploration
Source: Nat Commun. 2022 May 4;13:2450. doi: 10.1038/s41467-022-30069-1 (PMC9068924; doi:10.1038/s41467-022-30069-1)
Supplement: Supplementary file 3 — Description of Additional Supplementary Files [file 41467_2022_30069_MOESM3_ESM.pdf]

Description of Additional Supplementary Files

Supplementary Movie 1 | Behavioral exploration across re-aiming paradigm, example trials

In this video, we include trials from an example animal demonstrating reaches to A and B across the identified neural states. For trials after the pellet location has been switched, we have overlaid the previous A location in the video alongside the actual pellet location B. In particular, these videos are from Rat 3, with the following sessions/trials:

Video 1. Baseline, Trial 90, Reach to A
Video 2. Automatic, Trial 74, Reach to A
Video 3. Variable, Trial 34, Reach to A
Video 4. Variable, Trial 73, Reach to B
Video 5. Relearned, Trial 54, Reach to B
Video 6. Relearned, Trial 76, Reach to A
