# Supplementary material for: Transition from predictable to variable motor cortex and striatal ensemble patterning during behavioral exploration
Source: Nat Commun. 2022 May 4;13:2450. doi: 10.1038/s41467-022-30069-1 (PMC9068924; doi:10.1038/s41467-022-30069-1)
Supplement: Supplementary file 5 — Reporting Summary [file 41467_2022_30069_MOESM5_ESM.pdf]

nature portfolio

Corresponding author(s): Karunesh Ganguly

Last updated by author(s): Mar 17, 2022

Reporting Summary

Nature Portfolio wishes to improve the reproducibility of the work that we publish. This form provides structure for consistency and transparency in reporting. For further information on Nature Portfolio policies, see our Editorial Policies and the Editorial Policy Checklist.

Statistics

For all statistical analyses, confirm that the following items are present in the figure legend, table legend, main text, or Methods section.

n/a	Confirmed	
☐	☒	The exact sample size (*n*) for each experimental group/condition, given as a discrete number and unit of measurement
☐	☒	A statement on whether measurements were taken from distinct samples or whether the same sample was measured repeatedly
☐	☒	The statistical test(s) used AND whether they are one- or two-sided<br>*Only common tests should be described solely by name; describe more complex techniques in the Methods section.*
☒	☐	A description of all covariates tested
☐	☒	A description of any assumptions or corrections, such as tests of normality and adjustment for multiple comparisons
☐	☒	A full description of the statistical parameters including central tendency (e.g. means) or other basic estimates (e.g. regression coefficient) AND variation (e.g. standard deviation) or associated estimates of uncertainty (e.g. confidence intervals)
☐	☒	For null hypothesis testing, the test statistic (e.g. F, t, r) with confidence intervals, effect sizes, degrees of freedom and P value noted<br>*Give P values as exact values whenever suitable.*
☒	☐	For Bayesian analysis, information on the choice of priors and Markov chain Monte Carlo settings
☐	☒	For hierarchical and complex designs, identification of the appropriate level for tests and full reporting of outcomes
☐	☒	Estimates of effect sizes (e.g. Cohen's *d*, Pearson's *r*), indicating how they were calculated

Our web collection on statistics for biologists contains articles on many of the points above.

Software and code

Policy information about availability of computer code

Data collection	We recorded extracellular neural activity, including units and local field potentials (LFP), using an RZ2 system (Tucker-Davis Technologies) with Synapse software (v95). For 2/6 animals, behavior was video recorded using a side-view camera. For the remaining 4/6 animals, behavior was recorded using both top-down and side-view cameras. Three types of cameras were used: Microsoft LifeCams, which captured videos at 30Hz; Basler cameras, which captured videos at 75Hz; and Point Grey/FLIR cameras, which captured videos at 75-100Hz. Reach trajectories were captured from video using DeepLabCut41 v2.2 to track the center of the rat's paw.
Data analysis	Analyses were conducted using a combination of custom-written scripts and functions in MATLAB R2018B (MathWorks), along with functions from the EEGLAB (http://sccn.ucsd.edu/eeglab/) and the Chronux v2.12 (http://chronux.org/) toolboxes.

For manuscripts utilizing custom algorithms or software that are central to the research but not yet described in published literature, software must be made available to editors and reviewers. We strongly encourage code deposition in a community repository (e.g. GitHub). See the Nature Portfolio guidelines for submitting code & software for further information.

Data

Policy information about availability of data

All manuscripts must include a data availability statement. This statement should provide the following information, where applicable:

- Accession codes, unique identifiers, or web links for publicly available datasets
- A description of any restrictions on data availability
- For clinical datasets or third party data, please ensure that the statement adheres to our policy

The data used for analyses supporting the findings of this study are available from the corresponding author upon reasonable request. Source data are provided with this paper.

March 2021

Field-specific reporting

Please select the one below that is the best fit for your research. If you are not sure, read the appropriate sections before making your selection.

☒ Life sciences ☐ Behavioural & social sciences ☐ Ecological, evolutionary & environmental sciences

For a reference copy of the document with all sections, see nature.com/documents/nr-reporting-summary-flat.pdf

Life sciences study design

All studies must disclose on these points even when the disclosure is negative.

Sample size	No statistical methods were used to predetermine cohort sizes, but our sample sizes are similar to those reported in previous publications Lemke, et al. 2019, Li, et al. 2017, Santos, et al. 2015).
Data exclusions	Sessions which did not have both M1 and DLS units were not included in main analyses (Figure 3 onwards) due to insufficient data quality to warrant inclusion. Additionally, sessions in which video frames could not be aligned to neural timing were excluded from analysis, as detailed in the Methods section. No animals were excluded.
Replication	Linear mixed-effects models, with animal modeled as random effect on intercept and fixed effect for session type, were used to test the significance of differences across both behavioral and neural measures when comparing differences in group means. Thus, each animal was used as its own control to then replicate the findings across animals. A total of 6 animals were used, and for all neural analyses except for CCA, only one session of each session type per animal was used to preserve independence. Full details regarding each session and categorization into types are available in Figure 2e.
Randomization	Allocation was not random, however we varied the physical pellet locations of B across animals in order to ensure that locations A and B were not systematically at a central versus lateral position (n=2 central A with switch to lateral B and then n=4 lateral A with switch to central B). Covariate allocation was irrelevant, as both locations were easily reachable by the rat (i.e. one position was not harder than the other).
Blinding	Investigators were not blinded due to the experimental conditions being built into the training paradigm.

Reporting for specific materials, systems and methods

We require information from authors about some types of materials, experimental systems and methods used in many studies. Here, indicate whether each material, system or method listed is relevant to your study. If you are not sure if a list item applies to your research, read the appropriate section before selecting a response.

Materials & experimental systems

n/a	Involved in the study	
☒	☐	Antibodies
☒	☐	Eukaryotic cell lines
☒	☐	Palaeontology and archaeology
☐	☒	Animals and other organisms
☒	☐	Human research participants
☒	☐	Clinical data
☒	☐	Dual use research of concern

Methods

n/a	Involved in the study	
☒	☐	ChIP-seq
☒	☐	Flow cytometry
☒	☐	MRI-based neuroimaging

Animals and other organisms

Policy information about studies involving animals; ARRIVE guidelines recommended for reporting animal research

Laboratory animals	Adult male Long-Evans rats between 3 and 6 months old (n=9, 300-500g; Charles River Laboratories)
Wild animals	No wild animals were used in this study.
Field-collected samples	No field-collected samples were used in this study.
Ethics oversight	All procedures were in accordance with protocols approved by the Institutional Animal Care and Use Committee at the San Francisco Veterans Affairs Medical Center.

Note that full information on the approval of the study protocol must also be provided in the manuscript.
